# Supplementary material for: Conspecific and heterospecific cueing in shelter choices of Blaptica dubia cockroaches
Source: PeerJ. 2024 Mar 15;12:e16891. doi: 10.7717/peerj.16891 (PMC10946387; doi:10.7717/peerj.16891)
Supplement: Supplemental Information 1 — Estimates of fixed effects, expressed as relative risk ratios (RRR), for our selected multinomial multilevel logistic regression model for shelter choice (using the shelter with the conspecific cue as base outcome) in Experiment 1. Estimates of random effects are expressed as standard deviations. [file peerj-12-16891-s001.docx]

**Table S1**

Number of *dubia* roaches tested in Experiment 1 as a function of sex and trial. Three *dubia* roaches died during the study and so were removed from analyses (see text for details). In Experiment 1, each animal was tested six times under the same condition for a total of 558 observations (93 roaches x 6 trials).

|  | Sex | Trial | | | | | |
| --- | --- | --- | --- | --- | --- | --- | --- |
|  |  | 1 | 2 | 3 | 4 | 5 | 6 |
| Experiment 1 | Female | 46 | 46 | 46 | 46 | 46 | 46 |
|  | Male | 47 | 47 | 47 | 47 | 47 | 47 |
