# Supplementary material for: Conspecific and heterospecific cueing in shelter choices of Blaptica dubia cockroaches
Source: PeerJ. 2024 Mar 15;12:e16891. doi: 10.7717/peerj.16891 (PMC10946387; doi:10.7717/peerj.16891)
Supplement: Supplemental Information 2 — Estimates of fixed effects, expressed as relative risk ratios (RRR), for our selected multinomial multilevel logistic regression model for shelter choice (using the shelter with the conspecific cue as base outcome) in Experiment 1. Estimates of random effects are expressed as standard deviations. [file peerj-12-16891-s002.docx]

**Table S2**

Estimates of fixed effects, expressed as relative risk ratios (RRR), for our selected multinomial multilevel logistic regression model for shelter choice (using the shelter with the conspecific cue as base outcome) in Experiment 1. Estimates of random effects are expressed as standard deviations.

|  | Category | Effect | Estimate (RRR) | Robust SE | *Z* | *p* | 95%CI | |
| --- | --- | --- | --- | --- | --- | --- | --- | --- |
|  | |  |  |  |  |  | *LL* | *UL* |
| Fixed effects | | | | | | | | |
|  | Conspecific cue shelter | | | | | | | |
|  | | Base outcome | | | | | | |
|  | Control cue shelter | | | | | | | |
|  |  | Intercept | 0.177 | 0.033 | -9.42 | 0.000 | 0.124 | 0.254 |
|  | Out | | | | | | | |
|  | | Intercept | 0.433 | 0.059 | -6.17 | 0.000 | 0.332 | 0.565 |
| Random effects | | | | | | | | |
|  | Conspecific cue shelter | | | | | | | |
|  | Base outcome | | | | | | | |
|  | Control cue shelter | | | | | | | |
|  | | Roaches | 0.211 | 0.544 |  |  | 0.001 | 32.866 |
|  | Out | | | | | | | |
|  | | Roaches | 0.643 | 0.171 |  |  | 0.381 | 1.084 |

Note. N = 558 observations. CI = confidence interval; LL = lower limit; UL = upper limit.
