# Supplementary material for: Conspecific and heterospecific cueing in shelter choices of Blaptica dubia cockroaches
Source: PeerJ. 2024 Mar 15;12:e16891. doi: 10.7717/peerj.16891 (PMC10946387; doi:10.7717/peerj.16891)
Supplement: Supplemental Information 3 — Number of dubia roaches tested in Experiment 2 and Experiment 3 as a function of sex, group size and cue location. In Experiment 2, we used chemical cues from other dubia roaches. In Experiment 3, we used chemical cues from a different species, the death’s head cockroach, Blaberus craniifer. In both experiments, each animal was tested once for a total of 512 observations. [file peerj-12-16891-s003.docx]

**Table S3**

Number of *dubia* roaches tested in Experiment 2 and Experiment 3 as a function of sex, group size and cue location. In Experiment 2, we used chemical cues from other *dubia* roaches. In Experiment 3, we used chemical cues from a different species, the death’s head cockroach, *Blaberus craniifer*. In both experiments, each animal was tested once for a total of 512 observations.

|  | Sex | Group Size | Chemical cue location | |
| --- | --- | --- | --- | --- |
|  |  |  | Lighter shelter | Darker shelter |
| Experiment 2 | Female | 1 | 32 | 32 |
|  |  | 3 | 96 | 96 |
|  | Male | 1 | 32 | 32 |
|  |  | 3 | 96 | 96 |
| Experiment 3 | Female | 1 | 32 | 32 |
|  |  | 3 | 96 | 96 |
|  | Male | 1 | 32 | 32 |
|  |  | 3 | 96 | 96 |
