# Supplementary material for: Conspecific and heterospecific cueing in shelter choices of Blaptica dubia cockroaches
Source: PeerJ. 2024 Mar 15;12:e16891. doi: 10.7717/peerj.16891 (PMC10946387; doi:10.7717/peerj.16891)
Supplement: Supplemental Information 4 — Estimates of fixed effects, expressed as relative risk ratios (RRR), for our selected multinomial logistic regression model for shelter choice (using the shelter with the conspecific cue as base outcome) in Experiment 2. Estimates of random effects are expressed as standard deviations for rounds of testing when standardized. [file peerj-12-16891-s004.docx]

**Table S4**

Estimates of fixed effects, expressed as relative risk ratios (RRR), for our selected multinomial logistic regression model for shelter choice (using the shelter with the conspecific cue as base outcome) in Experiment 2. Estimates of random effects are expressed as standard deviations for rounds of testing when standardized.

|  | Category | Effect | Estimate (RRR) | Robust SE | *Z* | *p* | 95%CI | |
| --- | --- | --- | --- | --- | --- | --- | --- | --- |
|  | |  |  |  |  |  | *LL* | *UL* |
| Fixed effects | | | | | | | | |
|  | Darker shelter | | | | | | | |
|  | | Base outcome | | | | | | |
|  | Lighter shelter | | | | | | | |
|  |  | Cue | 36.023 | 14.949 | 8.64 | 0.000 | 15.971 | 81.250 |
|  |  | Intercept | 0.016 | 0.007 | -9.27 | 0.000 | 0.007 | 0.039 |
|  | Out | | | | | | | |
|  | | Cue | 9.110 | 4.776 | 4.21 | 0.000 | 3.261 | 25.453 |
|  | | Intercept | 0.041 | 0.019 | -6.91 | 0.000 | 0.017 | 0.202 |
| Random effects | | | | | | | | |
|  | Conspecific cue shelter | | | | | | | |
|  | | Base outcome | | | | | | |
|  | Control cue shelter | | | | | | | |
|  | | Rounds | 0.500 | 0.329 |  |  | 0.138 | 1.818 |
|  | Out | | | | | | | |
|  | | Rounds | 0.001 | 0.120 |  |  | -0.000 | 5.9e+75 |

Note. Cue = Conspecific cue location, N = 512 observations. CI = confidence interval; LL = lower limit; UL = upper limit.
