# Supplementary material for: Conspecific and heterospecific cueing in shelter choices of Blaptica dubia cockroaches
Source: PeerJ. 2024 Mar 15;12:e16891. doi: 10.7717/peerj.16891 (PMC10946387; doi:10.7717/peerj.16891)
Supplement: Supplemental Information 5 — Estimates of fixed effects, expressed as relative risk ratios (RRR), for our selected multinomial logistic regression model for shelter choice (using the shelter with the heterospecific cue as base outcome) in Experiment 3. [file peerj-12-16891-s005.docx]

**Table S5**

Estimates of fixed effects, expressed as relative risk ratios (RRR), for our selected multinomial logistic regression model for shelter choice (using the shelter with the heterospecific cue as base outcome) in Experiment 3.

|  | Category | Effect | Estimate (RRR) | Robust SE | *Z* | *p* | 95%CI | |
| --- | --- | --- | --- | --- | --- | --- | --- | --- |
|  | |  |  |  |  |  | *LL* | *UL* |
| Fixed effects | | | | | | | | |
|  | Darker shelter | | | | | | | |
|  | | Base outcome | | | | | | |
|  | Lighter shelter | | | | | | | |
|  |  | Cue | 14.321 | 11.053 | 3.45 | 0.001 | 3.155 | 65.009 |
|  |  | Intercept | 0.009 | 0.006 | -6.71 | 0.000 | 0.002 | 0.035 |
|  | Out | | | | | | | |
|  | | Cue | 1.410 | 0.581 | 0.83 | 0.404 | 0.629 | 3.161 |
|  | | Intercept | 0.114 | 0.037 | -6.76 | 0.000 | 0.061 | 0.214 |

Note. Cue = Heterospecific cue location. N = 512 observations. CI = confidence interval; LL = lower limit; UL = upper limit.
